# A database of georeferenced nutrient chemistry data for mountain lakes of the Western United States

**DOI:** 10.1038/sdata.2017.69

**Published:** 2017-05-16

**Authors:** Jason Williams, Stephanie G. Labou

**Affiliations:** 1 Washington State University, Department of Civil & Environmental Engineering, Pullman, Washington 99164, USA; 2 Washington State University, Center for Environmental Research, Education, and Outreach (CEREO), PACCAR, Pullman, Washington 99164, USA

**Keywords:** Element cycles, Limnology

## Abstract

Human activities have increased atmospheric nitrogen and phosphorus deposition rates relative to pre-industrial background. In the Western U.S., anthropogenic nutrient deposition has increased nutrient concentrations and stimulated algal growth in at least some remote mountain lakes. The Georeferenced Lake Nutrient Chemistry (GLNC) Database was constructed to create a spatially-extensive lake chemistry database needed to assess atmospheric nutrient deposition effects on Western U.S. mountain lakes. The database includes nitrogen and phosphorus water chemistry data spanning 1964–2015, with 148,336 chemistry results from 51,048 samples collected across 3,602 lakes in the Western U.S. Data were obtained from public databases, government agencies, scientific literature, and researchers, and were formatted into a consistent table structure. All data are georeferenced to a modified version of the National Hydrography Dataset Plus version 2. The database is transparent and reproducible; R code and input files used to format data are provided in an appendix. The database will likely be useful to those assessing spatial patterns of lake nutrient chemistry associated with atmospheric deposition or other environmental stressors.

## Background & Summary

In many temperate lakes, inputs of nitrogen and phosphorus strongly affect lake ecosystem structure and the capacity of lakes to provide ecosystem services to humans^1^. Growth of algae at the base of lake food webs is often limited by the supply of nitrogen or phosphorus^2,3^. Increasing the supply of these elements can therefore cause lake eutrophication, wherein lake nutrient concentrations and the rate of algal biomass growth increase^4^. Many undesirable ecosystem changes can occur during eutrophication, including reduced water transparency, harmful algal blooms, and reduced oxygen-rich habitat for fish and other organisms^1^.

In the United States (U.S.), one way humans have increased nitrogen and phosphorus inputs to lakes, and thereby increased the potential for lake eutrophication, is by increasing nitrogen and phosphorus atmospheric deposition rates. Anthropogenic emissions of reactive nitrogen (NO_x_ and NH_x_) associated with fossil fuel combustion, industrial nitrogen fixation, and agricultural activities have increased atmospheric nitrogen deposition rates to ecosystems across the country^5,6^. Human expansion of livestock grazing, dryland agriculture, mining, and use of phosphorus fertilizers have also increased dust mobilization and phosphorus content, and thereby increased atmospheric phosphorus deposition rates in some Western U.S. regions^7^.

In the Western U.S., there are thousands of lakes where anthropogenic atmospheric nutrient deposition has the potential to increase lake nutrient concentrations, stimulate algal growth, and cause eutrophication effects. Lakes most sensitive to these effects are typically those at high elevation, or with undeveloped watersheds, including lakes within National Parks, National Forests, and Wilderness areas. High elevation lakes with undeveloped watersheds (hereafter ‘mountain lakes’) are especially sensitive to atmospheric nutrient inputs because they often have nutrient concentrations near or below analytical detection limits, and have watershed characteristics that promote efficient flux of deposited nutrients into lakes^8,9^. There is increasing evidence that anthropogenic atmospheric nitrogen^10,11^ and phosphorus^7,12,13^ deposition has already altered at least some mountain lake ecosystems in the Western U.S.

However, the number and spatial distribution of Western U.S. mountain lakes altered by anthropogenic nutrient deposition is not clear. Assessing patterns at large spatial scales requires relating a spatially-extensive database of lake nitrogen and phosphorus chemistry data to deposition estimates, but no such database is currently available. Although many lake monitoring efforts have quantified nitrogen and phosphorus concentrations in mountain lakes, these disparate datasets have not been combined into a single publically-available database. The U.S. Environmental Protection Agency (USEPA) has conducted several relevant national-scale lake surveys, such as the 1984 Western Lakes Survey (WLS)^14^, and the 2007 and 2012 National Lake Assessments (NLA), each of which included some mountain lakes. Federal land management agencies such as the National Park Service (NPS), and U.S. Forest Service (USFS) also regularly sample mountain lakes in specific federal land units. Numerous university and government agency research projects have also examined nitrogen and/or phosphorus deposition impacts within specific regions. However, all of these data sources vary in terms of time span, sampling protocols, chemical species measured, documentation, data curation, and data accessibility, are stored in disparate locations, and have not previously been combined. Data harmonization, the process of integrating datasets generated using different sampling methods, levels of documentation, and conventions for naming, units, and other characteristics, can be challenging and labor-intensive, but is critical for addressing ecological questions at large spatial scales^15^.

**Figure 1:**
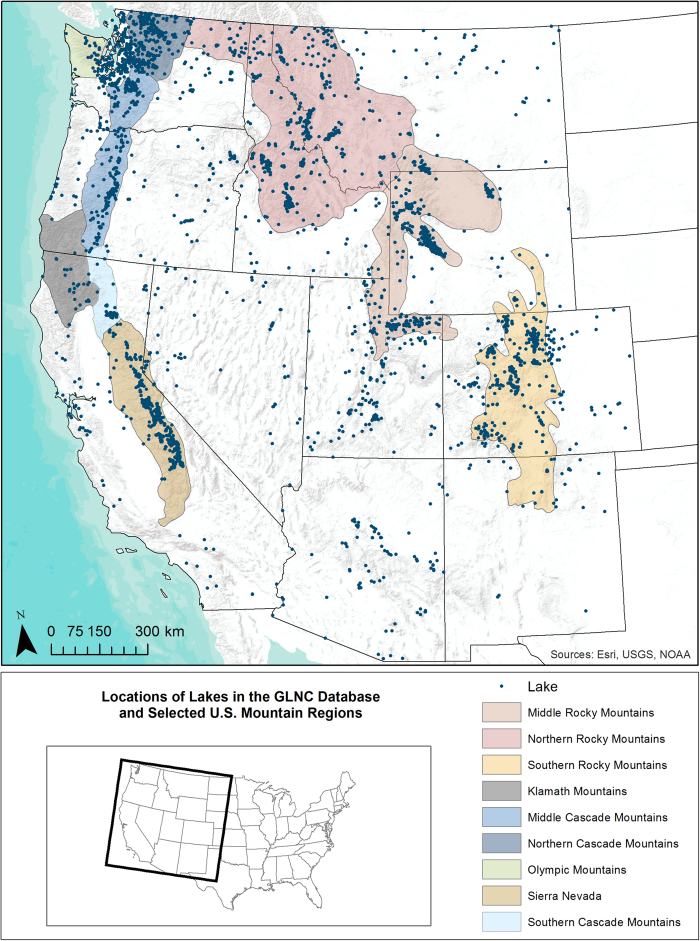
Locations of lakes in the GLNC database and selected mountain regions.

The objective of this study was to combine existing Western U.S. mountain lake nutrient chemistry datasets into a single database that can help assess deposition effects on mountain lakes and identify regional and national-scale data needs. Data obtained from federal agencies, researchers, and the literature were combined to create a georeferenced database with 148,336 nutrient chemistry results from 51,048 samples collected across 3,602 lakes in the Western U.S. states (Fig. 1). All data records in the database are georeferenced to individual lakes using a modified version of the National Hydrography Dataset (NHDPlus version 2). A shapefile delineating polygons for GLNC database lakes is included along with the chemistry database. The database was also constructed to be transparent and reproducible: data input files and R code used to format and combine data sources are provided as an appendix to the database.

## Methods

### Data acquisition and inclusion criteria

Because the primary motivation for constructing the GLNC database was to support assessment of atmospheric nutrient deposition effects on mountain lakes, data acquisition efforts targeted studies assessing atmospheric deposition effects on mountain lakes and data collected within Western U.S. federally protected lands (national parks, national forests, wilderness areas). Data from other lake types (lower elevation lakes, reservoirs) were included in the GLNC database only if they were included in a targeted dataset along with mountain lake data. All data sources included in the GLNC database are listed in Table 1. Water chemistry data were obtained from publically-available databases, through e-mail requests to researchers and federal agency staff, and from relevant peer-reviewed literature.

Data from the National Water Quality Monitoring Council Water Quality Portal (WQ Portal) (http://waterqualitydata.us/) were queried using the WQ Portal website on June 22, 2016. WQ Portal stores data collected through federal, tribal, state, and local water quality monitoring efforts. The query was constrained to selected nitrogen and phosphorus species from lakes and reservoirs in 11 western U.S. states (AZ, CA, CO, ID, MT, NV, NM, OR, UT, WA, WY). WQ Portal data include both mountain lakes and other lake types. Complete documentation of the query constraints and query output are provided in the database appendix.

At the time this database was constructed, WQ Portal did not contain all mountain lake water chemistry data collected by U.S. federal agencies. Data from the USEPA 1985 Western Lakes Survey were obtained from USEPA (Jason Lynch, personal communication). Data from the 2007 and 2012 USEPA National Lakes Assessments were obtained from the USEPA website (https://www.epa.gov/national-aquatic-resource-surveys/nla). To obtain relevant NPS and USFS data not in WQ Portal, e-mail requests were sent to agency staff. Data requests were sent to staff associated with NPS Inventory and Monitoring (I&M) networks in the Western U.S. (http://science.nature.nps.gov/im/). Data were provided by the NPS Rocky Mountain (ROMN), North Coast and Cascades (NCCN), Klamath (KLMN), and Sierra Nevada SIEN) monitoring networks. In addition, data requests were sent to USFS staff, who provided output of a specialized query of the Federal Land Manager Environmental Database (FED, http://views.cira.colostate.edu/fed/) in November 2015. FED is maintained by multiple federal agencies, and contains environmental data federal land managers use to assist assessment of air quality impairment in federal lands, including air quality impacts on aquatic ecosystems.

To obtain scientific literature data, publications assessing deposition effects on mountain lakes were targeted. A systematic search of peer-reviewed publications was not performed due to time and budget constraints. Data were obtained from the publication itself if possible, or by sending e-mail requests to publication authors.

Accessed data were included in the database if they met a minimum set of criteria developed based on the intended database application. Data were included if:

1. Samples had associated water chemistry data for one or more targeted elements (N,P). Si, chlorophyll a, and seston nutrient chemistry data were also included if they were provided along with N and P water chemistry data.
2. Data were derived from grab samples from any depth, or were depth-integrated samples collected by integrating water collected near the surface (within 0–2 m).
3. Sample depth information was available, either in categorical (i.e., ‘surface’, ‘epilimnion’, ‘lake bottom’) or quantitative (i.e., 1.5 m) form.
4. Sample date information was available, or at least sample month and sample year.
5. Samples met quality assurance requirements specified in the original data source; data flagged as ‘rejected’ or ‘invalid’ were excluded.
6. Data were derived from ‘regular’, field duplicate, or field triplicate samples. Results associated with field blanks, splits, and laboratory quality control samples were excluded.
7. The location of the associated lake could be verified through GIS research; a list of data records excluded based on GIS research is included in the database appendix.

Data were constrained to grab samples with depth information or near-surface integrated samples because depth and lake stratification strongly affect nutrient concentrations and the responsiveness of water chemistry to atmospheric pollutants. Samples collected from the lake surface, epilimnion, or lake outlet are typically used to investigate effects of atmospheric deposition on lake chemistry^16^. Samples integrated across the water column or taken from bottom waters may reflect fluxes of geologically-derived or historically-deposited nutrients from anoxic sediments rather than from recent atmospheric deposition inputs to the lake or watershed. Because the database is constrained based on the available depth information, but not on sample depth value, database users can subset the data most relevant to their research question.

### Workflow overview

**Figure 2:**
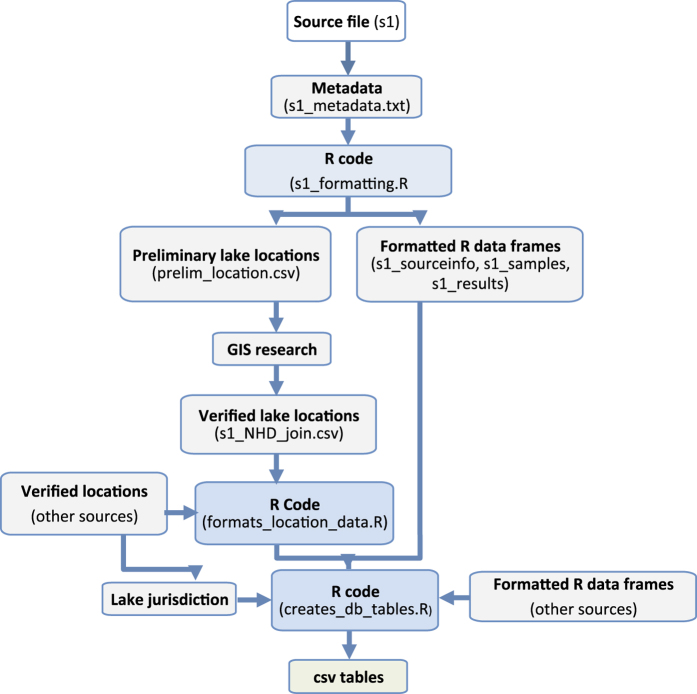
Workflow schematic. ***source file s1:*** data file(s) provided by the data source (typically.csv or.xlsx). ***s1_metadata.txt:*** a.txt file that describes the provenance of file s1, and includes relevant notes about s1 data, and s1 data processing. ***s1_formatting.R***: annotated R code file that uses data in source file s1 to populate structured R data frames, and generate a.csv file that will be read into ArcMap to confirm lake location information. **s1_prelim_location.csv:** a csv file with latitude and longitude information from s1. This lat/long information was read into ArcMap to georeference the lake location according to NHD Plus Version 2 ComID. **s1_NHD_join.csv:** a csv file with verified lake location (NHDPlus version 2 ComID), and associated research notes. **formats_location_data.R:** annotated R code file that reads and formats csv files generated through GIS research (sx_NHD_join.csv files). **creates_db_tables.R:** annotated R code file that combines and formats data to create database tables in csv file form. **csv tables:** GLNC database tables in csv form (see section 3.0 Summary of Data records for table descriptions).

**Figure 3:**
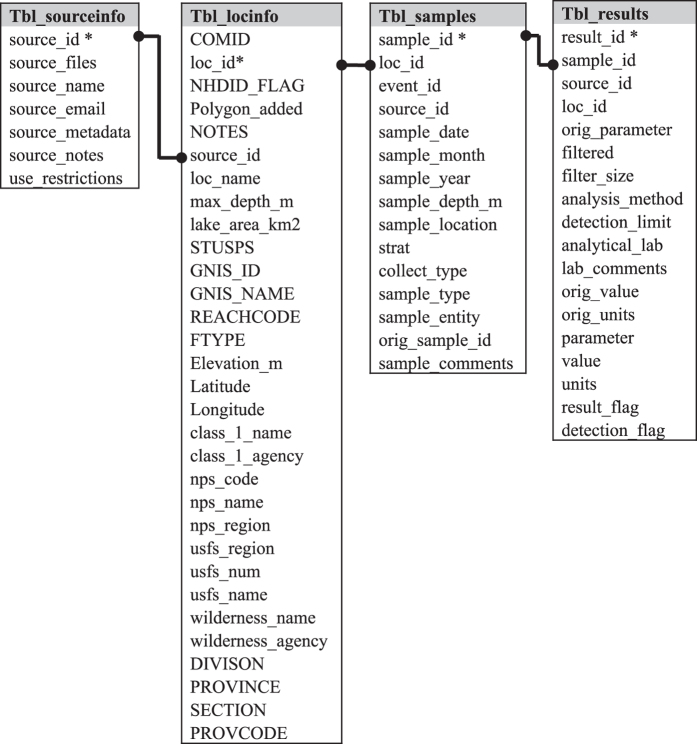
Relationships between database tables. Only selected relationships are marked by connector lines. Fields marked by * are primary keys. Table fields are defined in the database data dictionary.

To construct the database, relevant data from each data source were georeferenced, formatted, and placed into a defined database table structure. Raw data files varied widely in content and formatting, so each data source had to be processed individually. A standardized series of processing steps (Fig. 2) was applied to each source to generate a collection of associated data files and metadata, and ultimately to populate database tables containing information about the data source (Tbl_sourceinfo), geographic information (Tbl_locinfo), samples (Tbl_samples), and results (Tbl_results) (Fig. 3). Files generated during processing of each source are provided in the database appendix. The fields in each final table are described further below and in the database data dictionary. Our workflow was modelled after that described in Soranno *et al.*^15^, who described procedures for integrating multiple datasets into a reproducible georeferenced ecology database, as well as ‘lessons learned’ from undertaking such efforts^15^.

During processing, source data records were assigned ids in the GLNC database to help users trace data back to its original source, and to enable table joins. GLNC ids include an id for the data source (source_id), an id for each latitude/longitude point (loc_id), an id for each sample event (event_id), an id for each sample (sample_id), and an id for each analytical result (result_id). Ids assigned to lakes and samples by the original data source are also retained in the database.

Source data were also used to create an aggregated parameter field ‘parameter’ in Tbl_results that is intended to increase the utility of the database for data analysis. In some cases, different data sources used different names or units for the same parameter, or it was desirable to aggregate distinct but similar parameters to a single aggregated parameter. For example, differing names and units reported for dissolved nitrate were aggregated into a single parameter with a consistent name and units (NO_3__N_mgL). Sample ids, parameter names, parameter units, and parameter values in the original source data were also retained in the final data tables. A more detailed description of assigned ids, parameter aggregation, and fields and values in each data table is provided below the ‘Usage Notes’ section, and in the data dictionary provided with the database.

### Lake georeferencing

Data in the GLNC database were georeferenced using the National Hydrography Dataset (NHDPlusv2, http://www.horizon-systems.com/nhdplus/NHDPlusV2_home.php). NHDPlusv2 is a free suite of geospatial datasets developed to provide a nationally consistent source of geospatial hydrologic data and support water resources applications in the U.S. NHDPlusv2 includes polygons for U.S. lakes, and each lake polygon has a unique identification number (ComID) that can be used to access a wide array of associated lake attribute, elevation, and hydrologic information. Using a semi-automated process described in a file included with the database, ‘lake_georeferencing_procedure.doc’, lake latitude and longitude points in each data source were used to determine the ComID associated with each GLNC data record. Latitude and longitude points from each data source were spatially joined to NHDPlusv2 lake polygons in ArcMap, and then a series of procedures were implemented to verify that ComIDs identified through the spatial join were correct. Contributed data were excluded from the GLNC database if the location of the associated lake could not be verified (see inclusion criteria above). The shapefile included with the database (‘NHDPlusv2_waterbodies_modified’) includes all NHDPlusv2 waterbodies from NHD regions 10–18, and the column ‘GLNC_lake’ in the shapefile attribute table indicates lakes with data in the GLNC database.

Many lakes with data in contributed files did not have a lake polygon in the NHDPlusv2. NHDPlusv2 does not include polygons for all lakes present on USGS topographic maps, especially many small remote lakes in the Western U.S. To address this, a modified version of the NHDPlusv2 dataset was created during the georeferencing process. If a lake had water chemistry data from a contributed source file, but did not have a polygon in NHDPlusv2, a lake polygon was drawn based on USGS topographic maps if the lake’s location could be verified, and the new lake polygon was assigned a unique ComID. In the database shapefile and Tbl_locinfo, polygons drawn during lake location research are indicated by ComID values (ComID values⩽596). In Tbl_locinfo, added polygons are also indicated by a value ‘y’ in the column Polygon_added. Note that lake polygons drawn during lake location research only provide lake location information; they cannot be linked to other NHDPlusv2 information through ComID values.

### Lake jurisdiction

After lake georeferencing was completed for all data sources, the state, federal land unit (national park, national forest, wilderness, class I areas), and physiographic province associated with each lake was determined by spatially joining lake polygons and geospatial datasets with jurisdiction boundaries (Table 2). Jurisdictions listed in the GLNC database are specific to the federal land unit boundary geospatial datasets in Table 2. Over the time period associated with GLNC data, some discrete federal land units have been consolidated into a single administrative unit and new land units have been created. As a result, the federal land unit a lake was associated with at the time a sample was collected may not always correspond to the federal land that the lake was assigned to in the GLNC database. In some cases raw source files indicated the federal land unit a lake was located within. This information is retained in source files, but was not retained in the database. Some jurisdiction assignments in the GLNC database may also become incorrect in the future if federal land unit boundaries change further.

### Code availability

R code used to format source data and create database tables (Fig. 2) are provided in a database appendix along with the database on FigShare (Data Citation 1). Database tables were created using R Version 3.2.1. Output of the R command sessionInfo(), which indicates the R libraries and settings used by code is documented in the appendix file ‘R_sessioninfo.txt’ in the appendix. See usage notes about reproducibility for more information on running R code.

## Data Records

The GLNC database is provided within the zip folder ‘GLNC_March_2017.zip’ (Data Citation 1). A list and description of files associated with the database is provided in ‘GLNC_database_file_list.txt’. The folder includes csv files for each database table (Tbl_sourceinfo.csv, Tbl_lakeinfo.csv, Tbl_samples.csv, Tbl_results.csv), and a lake polygon shapefile with ComIDs corresponding to those in Tbl_lakeinfo.csv (NHDPlusv2_waterbodies_modified). Field names and definitions for each table are provided in a data dictionary (GLNC_data_dictionary.doc), and in xml metadata for each table formatted using the Ecological Metadata Language (EML). In addition, the folder contains two csv files created by joining data from database tables. GLNC_data_long_format.csv contains data from all four tables, with one row per result, and GLNCdata_by_sample.csv contains data from all four tables, with one row per sample. See the ’Usage Notes’ section for additional information about GLNCdata_by_sample.csv. Creation of all 6 csv files is documented in R code file ‘creates_db_tables.R’ in the database appendix.

The database folder ‘GLNC_appendicies_March2017’ (Data Citation 1) includes files associated with each data source depicted in Fig. 2 (source data, metadata, R code used to format source data, and output of R code files). A list and description of files within this folder is provided in GLNC_database_file_list.txt.

The GLNC database includes 148,336 chemistry results from 51,048 samples collected across 3,602 lakes in the Western U.S. The number of lakes in the database within federal land unit boundaries and within selected U.S. mountain regions are listed in Table 3 and Table 4, respectively. Effects of atmospheric nitrogen deposition on lakes are often evaluated using lake NO_3_-N for nitrate leaching effects^10^, and the mass ratio of dissolved inorganic nitrogen to total phosphorus (DIN:TP) to assess impacts on phytoplankton nutrient limitation^10,17^. The number of lakes with data for these parameters are summarized in Tables 3 and 4.

## Technical Validation

Four types of technical validation procedures were performed. First, a systematic review of R code written to format source data and create data tables was performed. All R code was written by one person (Jason Williams) and independently reviewed by a second (Stephanie Labou) to verify code performed as intended, and that annotations within code files were complete and informative. Second, checks were performed using R to identify and remove data that were duplicated across or within data sources. Some water samples were included in multiple data sources; these cross-source duplicates were identified and removed using procedures documented in the R code file ‘creates_db_tables.R’ in the database appendix. There were also some duplicate records within WQ Portal data derived from the USGS National Water Information System (NWIS). In some cases, results from the same sample were reported multiple times using multiple units; one was the original reported by the laboratory, and another was the same result converted to different units through a calculation procedure. These cases were identified, original results were retained, and calculated results were excluded. Checks and exclusions specific to WQ Portal data are documented in ‘s3_formatting.R’ in the database appendix.

Third, a series of QA/QC checks were performed on database tables. For each data source, a subset of data—5% of unique samples for sources with less than 1,000 samples, 1% for those with less than 10,000 samples, and 0.05% for those with over 10,000 samples—were compared between raw source data and final data tables to check for data processing errors. In addition, a series of simple spot-checks were performed on information in Tbl_location to verify jurisdiction information obtained through spatial joins was correct. For example, we checked if data provided by NPS were from lakes determined to be within NPS boundaries based on spatial joins. Fourth, a draft version of the database was provided to data contributors, who were asked to review and spot-check data derived for their contribution, although only a few contributors conducted these spot checks and provided a response. Database and R code revisions were made as needed during each technical validation step.

## Usage Notes

### Ids

Four types of ids were assigned to data records to facilitate data tracking and table joins.

A *source_id* is a unique identification numbers given to each data source (ex: ‘s1’ for source 1) (Table 1). The source_id field is present in all GLNC csv files (Fig. 3).

A *loc_id* is a unique identifier for a spatial point (latitude/longitude) associated with a sample. Each loc_id was constructed as source_id+lk+orig_lat+orig_long (ex: ‘s3_lk_31.3988883_-111.0881933), where orig_lat and orig_long are the latitude and longitude in units provided in the original data source. The loc_id field is present in Tbl_samples.csv, Tbl_results.csv, and Tbl_lakeinfo.csv.

An *event_id* is a unique identifier for a sample collection event. Each event_id was constructed as loc_id+sample_date (ex: ‘s3_lk_31.3988883_-111.0881933_2003-09-12’); each event_id indicates a unique combination of source_id, location, and date. The event_id field is present in Tbl_samples.csv.

A *sample_id* is a unique identifier for a sample. Each sample_id was constructed as: event_id+sample_number (ex: ‘s3_lk_31.3988883_-111.0881933_2003-09-12_1’), where sample number indicates the nth sample collected within an event_id. Each sample_id represents a unique combination of event_id, sample_depth_m, and sample_type. Original and field duplicate samples within an event_id were assigned different sample_ids. Note that sample_id values are in 1:1 relationship with the sample id assigned by the source, if provided (orig_sample_id in Tbl_samples.csv). Field duplicate samples are marked as ‘fdup’ in the ‘sample_type’ column of Tbl_samples.csv. The sample_id field is present in Tbl_samples.csv, and Tbl_results.csv.

A *result_id* is a unique identifier for each analytical result within a sample_id. Each result_id was constructed as: sample_id+r+result_number (ex: ‘s3_lk_31.3988883_-111.0881933_2003-09-12_1_r1)’, where sample number indicates the nth result associated with a sample. The result_id field is present in Tbl_results.csv.

### ComIDs

The ComID field in Tbl_locinfo.csv corresponds to the ComID field in GLNC_waterbodies_modified.shp. In cases where a lake in the GLNC database was included in NHDPlusv2, the ComID value is the same as that in NHDPlusv2, and can be used to join to NHDPlusv2 hydrologic data for the Western U.S. (NHDPlusv2 regions 10–18). In cases where a lake in the GLNC database was not included in NHDPlusv2, the ComID value (1–596) is unique to the GLNC database, and cannot be used to join to hydrologic data in NHDPlusv2.

### Latitude and longitude

In addition to being georeferenced through NHDPlusv2 ComID, lake location information is incorporated into Latitude and Longitude and loc_id fields within Tbl_locinfo. Latitude and Longitude fields are the latitude and longitude at the center point of the NHDPlusv2 lake polygon for the corresponding lake, using the NHDPlusv2 geographic projection (NAD83 Albers). In addition, latitude and longitude information from the original data source are retained within the loc_id field. Coordinates within the loc_id field do not represent coordinates of the lake center; they represent the location type (sample location, lake center, lake outlet, etc.) for which the data source recorded coordinates. Users should review source documentation if the source latitude and longitude data are of interest.

### Parameters

The GLNC database includes an aggregated parameter field ‘parameter’ in Tbl_results.csv that was created to increase the utility of the database. In some cases data sources used different names or units for the same parameter. For example, dissolved nitrate was reported as μmols NO3/l, mg NO3-N/l, μeq NO3/l, and mg NO3/l, among other formats. All nitrate results were aggregated to a single parameter ‘NO3_N_mgL’ with common units (mg N/L). In Tbl_results.csv, the ‘orig_parameter’, orig_units’, and ‘orig_value’ fields retain the values reported in the original data source. The ‘parameter’, ‘units’, and ‘value’ fields contain aggregated parameters, units, and values. The appendix file ‘parameter_conversion.csv’ documents how source data were used to create aggregated parameters. The GLNC data dictionary also includes values and associated definitions for the ‘parameter’ field in Tbl_results.csv. The number of lakes where data are available for each parameter is presented in Table 5.

In addition, in some cases it was desirable to aggregate distinct but similar parameters to a single aggregated parameter. For example, some sources reported nitrate plus nitrite data, whereas others reported nitrate data. Both of these original parameters were aggregated into a single parameter in Tbl_results.csv (‘NO3_N_mgL’) because nitrite is typically a very small fraction of nitrate plus nitrite. Similarly, results reported as NH3 and NH4 were aggregated into the single parameter NH4_N_mgL, and different analytical forms of phosphate were aggregated to ‘PO4_P_mgL’. All aggregation procedures are documented in the appendix file ‘parameter_conversion.csv’. Original parameter, units, and values provided by each data source are also retained in Tbl_results

Note that the parameter aggregation performed to create the ‘parameter’ field in Tbl_results.csv affects ‘GLNC_data_by_sample.csv’. For some sample_ids, there are multiple result_ids for the same parameter. For example, if phosphate was quantified both as orthophosphate and using another analytical method from the same sample, there are two result_ids for parameter ‘PO4_P_mgL’ within a sample_id. This means that converting data to wide format (one row per sample) requires handling these multiple results by taking an average, selecting one at random, or other approaches. GLNC_data_by_sample.csv was created by taking an average in these cases.

### Result flags, detection limits, and detection flags

The ‘result_flag’ field of Tbl_results.csv retains flags associated with an analytical result from the original data source. The ‘detection_flag’ field in Tbl_results.csv was created by interpreting the ‘result_flag’ field, and has a value of ‘bd’ if the data source flagged the result as below detection, ‘undetermined’ if no flag information was provided, and blank if flag information was provided but a result was not flagged as below detection. The ‘detection_limit’ field in Tbl_results.csv is the detection limit value from the data source (if provided), converted to aggregated parameter units. In some cases a result value (either ‘orig_value’ or ‘value’ fields) is less than the detection limit, but the detection flag column is blank because the result was not flagged as below detection in the original data source. Database users must use information in Tbl_results.csv to decide if and how to identify and address results that are below detection limits. Many data sources did not include detection limit information.

### Sample depth information

The Tbl_samples.csv field ‘sample_depth_m’ includes quantitative sample depth information, and the field ‘strat’ includes categorical sample depth information. The ‘sample_depth_m’ field is populated with a numeric value in meters if a sample depth measurement was provided in the data source. The ‘strat’ column is a categorical description of sample depth, which may describe the sample depth in terms of lake temperature zonation (ex: ‘EPLZ’=epilimnion, HYPZ=’hypolymnion’), or other categories (‘WTRS’=water surface, ‘WUBT’=water unit bottom). Definitions for all values of the ‘strat’ field are included in the GLNC data dictionary. Some data sources included information for only one of ‘sample_depth_m’ or ‘strat’, and some included both. Database users must use these fields to identify data records needed for their specific application.

### Time series information

The GLNC database was constructed to assess spatial rather than temporal patterns, but could be used for some temporal analyses. The database could be used to assess chemistry patterns across sample dates or sample years within a specific lake (ComID), but is not well-suited for shorter term (within day) temporal analyses. To assess patterns across dates or years within a specific lake (ComID), database users would need to evaluate results associated with unique sample_date values within a ComID of interest, while considering other relevant parameters (sample depth, sample fraction, parameter, etc). Because ComIDs can be located spatially using the shapefile provided with the GLNC database, or using NHDPlusv2, spatial and temporal data can also be linked.

However, assessing within-day time series information would require consulting source data files because the database does not include a field documenting sample time. However, our methods for constructing values in the sample_id field for each source were designed to ensure all values of sample_id in the database correspond to a unique sample. Additionally, we used source-specific unique sample IDs to construct the sample_id field when such information was provided in the source data. Users interested in within-day time series data would need to evaluate source data files to identify time series data. Sources s3, s11, s14, s17, and s18 included sample time information within source files read into R for formatting.

### Linking with atmospheric deposition data

Nitrogen and phosphorus deposition data are not included in the database because a variety of modelled deposition data sources are available, and data sources vary in terms of pollutants included, time period, spatial scale, and model performance. We leave it to users to determine the deposition data source most appropriate for specific applications. Several different types of publically-available atmospheric deposition GIS grids for nitrogen and other pollutants are available from the National Atmospheric Deposition Program (http://nadp.sws.uiuc.edu/data/annualmaps.aspx). Phosphorus deposition measurements and model data are available in the scientific literature^18–20^.

### Reproducibility

The GLNC database tables are fully reproducible; appendices include all source files and R code used to create the tables. R scripts used to develop the GLNC database utilize various R packages. As a result, running the GLNC R scripts and reproducing data tables exactly also requires utilizing the same R packages. A ‘snapshot’ of the R packages used to create the scripts has been saved using packrat (https://rstudio.github.io/packrat/, version 0.4.8–1), and is provided in the appendices. Instructions for running accessing these package dependencies while running GLNC R scripts are included in a README.txt file in the database appendices.

## Additional Information

**How to cite this article:** Williams, J. & Labou, S. G. A database of georeferenced nutrient chemistry data for mountain lakes of the Western United States. *Sci. Data* 4:170069 doi: 10.1038/sdata.2017.69 (2017).

**Publisher’s note:** Springer Nature remains neutral with regard to jurisdictional claims in published maps and institutional affiliations.

## Supplementary Material



## Figures and Tables

**Table 1:** Data sources.

**Source ID**	**Region**	**Origin or Reference**	Number of Lakes*	**Time Period**
s1	Sierras	^21^	50	2007–2008
s2	Rockies	^22^	5	2010
s3	11 Western states†	WQ Portal	1,105	1964–2016
s4-s10	USFS regions 1–6	FED Database	1,163	1973–2012
s11	Rockies	NPS ROMN Network Water Quality Database	49	1975–2004
s12	Rockies	^23^	39	2006
s13	Sierras	NPS SIEN Network mountain lake monitoring data	76	2008–2013
s14	Klamath mountains	NPS KLMN mountain lake monitoring data	35	2013
s15	Cascades	USGS	90	1989–1999
s16	Cascades	^24^	10	2009–2010
s17	Cascades, Olympics	NPS NCCN mountain lake monitoring data	184	1988–2015
s18	Western US	Western Lakes Survey	749	1985
s19	11 Western States†	National Lakes Assessment (2007)	298	2007
s20	Western US	WACAP database^25^	10	2003–2005
s21	Rockies	^26^	6	2008–2012
s22	Cascades	^27^	13	2013–2014
s23	Rockies	J. Brahney, personal communication	29	2009–2010
s24	Rockies	^28^	6	2011–2012
s25	11 Western States†	National Lakes Assessment (2012)	323	2012
NOTE: all data source files used to create the database are included in the GLNC database appendix.				

*number of lakes within the data source with data that met the criteria listed above; note that some lakes are included in multiple data sources.

†The original data sources includes data for the contiguous U.S, but only data from 11 Western U.S. states were incorporated into the GLNC database.

**Table 2:** Geospatial data used to determine lake jurisdiction.

**Geospatial Dataset**	**Source Description**
Federal Class I area boundaries	shapefile provided by Drew Bingham (NPS) 6-6-16.
NPS Boundaries	shapefile obtained from NPS Integrated Resource Management Applications (IRMA) portal (https://irma.nps.gov/Portal) 2-12-2015
USFS Boundaries	shapefile dated 2-9-16 obtained from http://data.fs.usda.gov/geodata/
Wilderness Boundaries	Shapefile dated 10-12-15 obtained from the Wilderness Institute http://www.wilderness.net/NWPS/geography
Physiographic provinces	shapefile obtained from http://water.usgs.gov/GIS/metadata/usgswrd/XML/physio.xml
State boundaries	Shapefile obtained from the US Census (cb_2015_us_state_5m) in June 2016 https://www.census.gov/geo/maps-data/data/cbf/cbf_state.html
Elevation	Hydrodem digital elevation models from the NHDPlusv2

**Table 3:** Number of GLNC lakes with NO_3_ and DIN:TP data by federal land unit jurisdiction.

**Jurisdiction**	**GLNC Lakes**	**Lakes with NO**_**3**_**-N data**	Lakes with DIN:TP data*
NPS	556	486	144
USFS	2,117	1,896	774
Wilderness†	3	3	1
other	926	481	335
Total protected	2,676	2,385	919
Grand Total	3,602	2,866	1,254
Note: 749 lakes with DIN:TP data derive from the 1985 Western Lake Survey.			

*number of lakes where NO_3_-N, NH_4_-N, and TP were all quantified from the same water sample.

†To prevent double-counting, the wilderness category includes only lakes in Wilderness areas that are not within national parks or national forest.

**Table 4:** Number of GLNC lakes with NO_3_ and DIN:TP data for selected mountain regions.

Mountain Region*	**GLNC Lakes**	**Lakes with NO**_**3**_**-N data**	**Lakes with DIN:TP data**†
Southern Rocky Mountains	424	383	211
Middle Rocky Mountains	508	462	164
Northern Rocky Mountains	573	524	208
Klamath Mountains	30	27	23
Southern Cascade Mountains	70	45	27
Northern Cascade Mountains	355	263	90
Olympic Mountains	23	17	15
Sierra Nevada	432	425	139

*regions were defined using physiographic section or province (Table 2).

†number of lakes where NO_3_-N, NH_4_-N, and TP were all quantified from the same water sample.

**Table 5:** GLNC database parameters and the number of lakes with associated data.

**Parameter**	**Description**	**Units**	**Number of Lakes**
NO3_N_mgL	nitrate-N	mg N/L	2,866
NH4_N_mgL	ammonium-N	mg N/L	2,554
TP_mgL	total phosphorus	mg P/L	2,041
PO4_P_mgL	phosphate P	mg P/L	1,841
Si_mgL	dissolved silica	mg Si/L	1,120
TNH4_N_mgL	total ammonium N	mg N/L	826
TDP_mgL	total dissolved phosphorus	mg P/L	752
chla_ugL	chlorophyll a	ug/L	714
TN_mgL	total nitrogen	mg N/L	672
TNO3_N_mgL	total nitrate N	mg N/L	468
TPO4_P_mgL	total phosphate P	mg P/L	365
TDN_mgL	total dissolved nitrogen	mg N/L	263
KN_mgL	dissolved Kjeldahl nitrogen	mg N/L	197
seston_N_mgL	seston N	mg N/L	165
seston_P_mgL	seston P	mg P/L	165
NO2_N_mgL	nitrite-N	mg N/L	136
TON_mgL	total organic nitrogen	mg N/L	64
DON_mgL	dissolved organic nitrogen	mg N/L	55
TKN_mgL	total Kjeldahl nitrogen	mg N/L	54
seston_NP	Seston N:P mass ratio	mass ratio	38
TN:TP_mass	TN:TP mass ratio	mass ratio	32
seston_N_mgg	sestion N	mg/g	22
seston_P_ppb	seston P	ppb	18
TIN_mgL	total inorganic nitrogen	mg N/L	12
TOP_mgL	total organic phosphorus	mg P/L	12
TIP_mgL	total inorganic phosphorus	mg P/L	10
DIN_mgL	dissolved inorganic nitrogen	mg N/L	6
DOP_mgL	dissolved organic phosphorus	mg P/L	5
